# The parkour tic-tac action versus the drop jump as part of complex training within the strength and conditioning programme of highly-trained youth basketball players

**DOI:** 10.1371/journal.pone.0315013

**Published:** 2024-12-19

**Authors:** Mark David Williams, Jorge Arede, Aiden Griggs, Jason Moran

**Affiliations:** 1 School of Sport, Rehabilitation and Exercise Sciences, University of Essex, Colchester, Essex, United Kingdom; 2 School of Psychology and Sport Science, Anglia Ruskin University, Cambridge, Cambridgeshire, United Kingdom; 3 School of Education, Polytechnic Institute of Viseu, Viseu, Portugal; 4 Department of Sports Sciences, Exercise and Health, University of Trás-os-Montes and Alto Douro, Vila Real, Portugal; 5 Research Center in Sports Sciences, Health Sciences and Human Development, CIDESD, Vila Real, Portugal; RCCS Istituto Auxologico Italiano, ITALY

## Abstract

This study aimed to examine the effects of two different complex training protocols on physical performance in highly-trained youth basketball players. Fourteen adolescent players participated in twice-weekly sessions over eight weeks, following either the Drop Jump protocol (n = 7) or the Tic-tac protocol (n = 7), performing 1–3 sets of 8–9 exercises. Physical performance was assessed before and after the intervention using jumping tests (CMJ, squat, 10–5 hop jumps), change-of-direction speed (5-10-5), sprinting (0–20 meters), and muscular strength (isometric midthigh pull) tests. The intraclass correlation coefficient of within subjects measures was 0.95. Results showed no significant fixed effects for group or time on performance variables (*p* > 0.05), with greater variance attributed to measurements rather than group differences. The interindividual response to training was highly variable, contingent on the performance outcome. These findings suggest that the parkour-based Tic-tac protocol can be included in strength and conditioning programs for youth basketball players to enhance sport-specific actions. However, to improve physical performance in young team-sport athletes, it is crucial to address the individual needs of each athlete. This includes acknowledging the highly individualised responses to training stimuli.

## Introduction

Strength and conditioning (S&C) training has become an integral component of the development of youth athletes [1–4]. This corresponds with a growing body of scientific evidence relating to resistance training in youth populations [3, 5], and the publication of position statements, from organisations such as the United Kingdom Strength and Conditioning Association and the National Strength and Conditioning Association, advocating for the benefits of such training in children and adolescents [6–8]. Accordingly, within youth athletic development models, such as the Youth Physical Development model [7], resistance training and other S&C-based activities (e.g., plyometric exercise) have been promoted as a means to enhance the physical capabilities of young athletes to better prepare them for the demands of organised sports [5, 9, 10].

One of the central aims of athletic development models is to enhance physical fitness qualities. In turn, this helps offset the risks associated with early single-sport specialisation, which involves a year-round commitment to a single sport in youth [11, 12]. Through exposure to high volumes of intensive training, single sport specialisation is purported to increase the risk of injury [13, 14]. Moreover, single sport specialisation may disrupt motor development and limit the learning of broad motor skills and movement capabilities [15]. To mitigate against such concerns, athletic models propose a systematic approach to training that aims to contribute to increased levels of motor competence and neuromuscular capabilities that, in turn, may reduce risk factors for injury [8, 9, 16]. Within this approach, the use of S&C activities may increase levels of muscular strength and motor skill performance beyond a level that could be achieved through growth and maturation alone [3, 17]. Accordingly, there has been an increased implementation of S&C training within youth sports. However, S&C coaches of youth athletes tend to place greater emphasis on developing resistance training competencies over those related to linear speed and agility [1]. Consequently, despite the benefits of resistance training, such training may be limited in terms of the breadth of movement skills to which the developing young athlete is exposed [18].

In addition to the above, the extent to which youth athletes are physically prepared for their sport may not be optimal. For example, in the sport of basketball, which requires high volumes of multidirectional movements and jumping actions, S&C programmes have been suggested to lack of specificity [19]. Specificity is a core principle within S&C training [20, 21] though within youth athletic development, training that is more general in nature is typically recommended, with progressions to more advanced training based upon the competency of the individual athlete [3, 22]. Nonetheless, for training to adequately prepare youth athletes for the rigours of their chosen sports, the content of the S&C training must be sufficient to account for the specific characteristics of those sports, while also meeting the individual developmental needs of each athlete.

Like adult basketball players, youth players are required to execute repeated high intensity efforts including vertical jumps, short distance sprints and changes of direction on the court [23, 24]. Proficiency in such movements has been found to be a differentiating factor between selected and non-selected players for a youth national basketball team [23], therefore, S&C training that targets all the physical capabilities required in basketball would appear necessary for optimal development. However, in accordance with the principle of *specific adaptation to imposed demands* (SAID), which holds that the body will only adapt to the stress being placed upon it [25], high-school basketball players have been found to display specific adaptations in response to different S&C training programmes [26]. For example, participants following a change of direction-focused programme significantly improved performance in a timed 10-m “zig-zag” test but did not significantly improve in measures of vertical jump performance compared to plyometric and strength training groups that did [26]. Accordingly, given the breadth of the physical requirements of basketball, obvious challenges exist for S&C coaches, especially when programming time is a constraint to optimal performance [27, 28].

Providing a solution to the above dilemma is the use of “complex training”, which combines heavy loads with lighter loads in two biomechanically similar movement patterns [29, 30]. In addition to its time-efficiency, complex training is also understood to create conditions of post-activation potentiation (PAP) for the subsequent exercise which, owing to increased motor neuron excitability, facilitates greater levels of force production [30]. However, while studies [29, 31] have shown the complex method to be effective in improving physical characteristics in youth basketball players, the exercises utilised in the “complex pair” appear to be limited to jumps occurring within the sagittal plane, which may not necessarily meet the demands of basketball, a sport which requires high-intensity actions in the frontal plane also [19].

Responses to training programmes in athletic populations may be difficult to detect, especially when the learning of new motor skills is necessary [32]. When aiming to improve skill acquisition and performance, coaches can provide athletes (learners) with clear instructions, such as the optimal technique to use, or they can design learning scenarios that encourage exploration of different movement scenarios [33]. In this regard, training has long been influenced by models rooted in pedagogy and sports psychology, relying on external guidance from coaches to instruct the performer towards a desired technical model [34]. However, more recently, there has been an emergence of models based on dynamic systems and biology [33]. This approach views the learner as a complex biological system composed of different parts that are independent, but which interact with one other. Thus, emphasis is placed primarily on changes in state over time rather than on entirely stable states [35]. Based on these assumptions, parkour—an activity requiring performers to travel between two points as quickly and as efficiently as possible while traversing obstacles and navigating varied surfaces [36, 37]—been proposed as an alternative method for developing movement capabilities and agility in team sport athletes, including young basketball players [36, 38].

Strafford et al. [36] propose using parkour to develop diverse movement capabilities based upon ideas from the Athletic Skills Model (ASM) [39, 40], a contemporary model for athletic development that incorporates principles from the ecological dynamics framework for motor learning and behaviour. The ASM introduces the notion of *donor sports*, suggesting that movement skills and action capabilities developed in one sport can be transferred to another, or *target sport* [36, 39]. From this perspective, the athletic pursuit, parkour, has been suggested to be an effective supplementary activity for young basketball players, offering diverse movement solutions that align more closely with the unpredictable environments and movement demands encountered in basketball games [18]. To date, however, there has been very little scientific evidence to support the donor sport concept meaning that more is required to clarify its effectiveness and programming potential for basketball coaches. Previously, Williams et al. [41] examined the use of parkour on the physical capabilities of youth basketball players. In the 8-week intervention study, which compared a parkour-based warm-up with a conventional neuromuscular training-based warm-up, no significant between-group differences were found in the preadolescent participants in test measures that included both vertical jumping and sprinting as well as a timed obstacle course, suggesting that parkour was as effective as typical S&C-based exercises [41]. In another study, Williams et al. [42] revealed that significantly greater maximal acceleration was produced in the parkour-based tic-tac action compared the S&C-based drop jump and the basketball lay-up shot in adolescent basketball players. However, no studies have examined the use of parkour-style activities in adolescent basketball players as part of a structured S&C programme, specially incorporating the use of complex training.

Based on such a lack of knowledge regarding the effectiveness and application of the donor sport concept, the aim of the present study was to examine the effects of two different complex training interventions, implemented within the normal strength and conditioning programme of talented adolescent basketball players, on measures of force, speed, and jumping capabilities. Specifically, this study aimed to evaluate the efficacy of the tic-tac jumping action when integrated within the training regimen of youth basketball players, and its potential to enhance specific performance outcomes.

## Materials and methods

### Study design and participants

Physical performance test data from highly-trained youth basketball players were analysed to compare the effects of two training interventions over an 8-week period. Male participants were from the same under-18 basketball academy, which is part the talent pathway within Basketball England, the national governing body for English basketball. Players within this structure complete the same fulltime basketball programme alongside their studies, that includes at least two structured S&C-based training sessions per week, and a competitive game against other academies across the country. Initially, sixteen under-18s players, were included in this study. However, due to unforeseen circumstances, one participant from each of the intervention groups were unable to partake in the post-intervention testing. Therefore, a total of 14 participants (age 17.2 ± 0.58 years; stature 188 cm ± 4.2; mass 77.86 kg ± 8.82) were included. All participants had at least six months experience of S&C training. Participants were randomly assigned to either the drop jump (DJ) or the tic-tac (TT) group by the by the 3^rd^ author of the study, while the other authors were blind to the participants’ groups. Ethical approval to use anonymised data for the study was granted by the institutional research ethics committee at the authors’ university, in accordance with the latest version of the Declaration of Helsinki. Data was accessed after approval had been obtained on 6^th^ February 2024.

### Training programme

**Fig 1:**
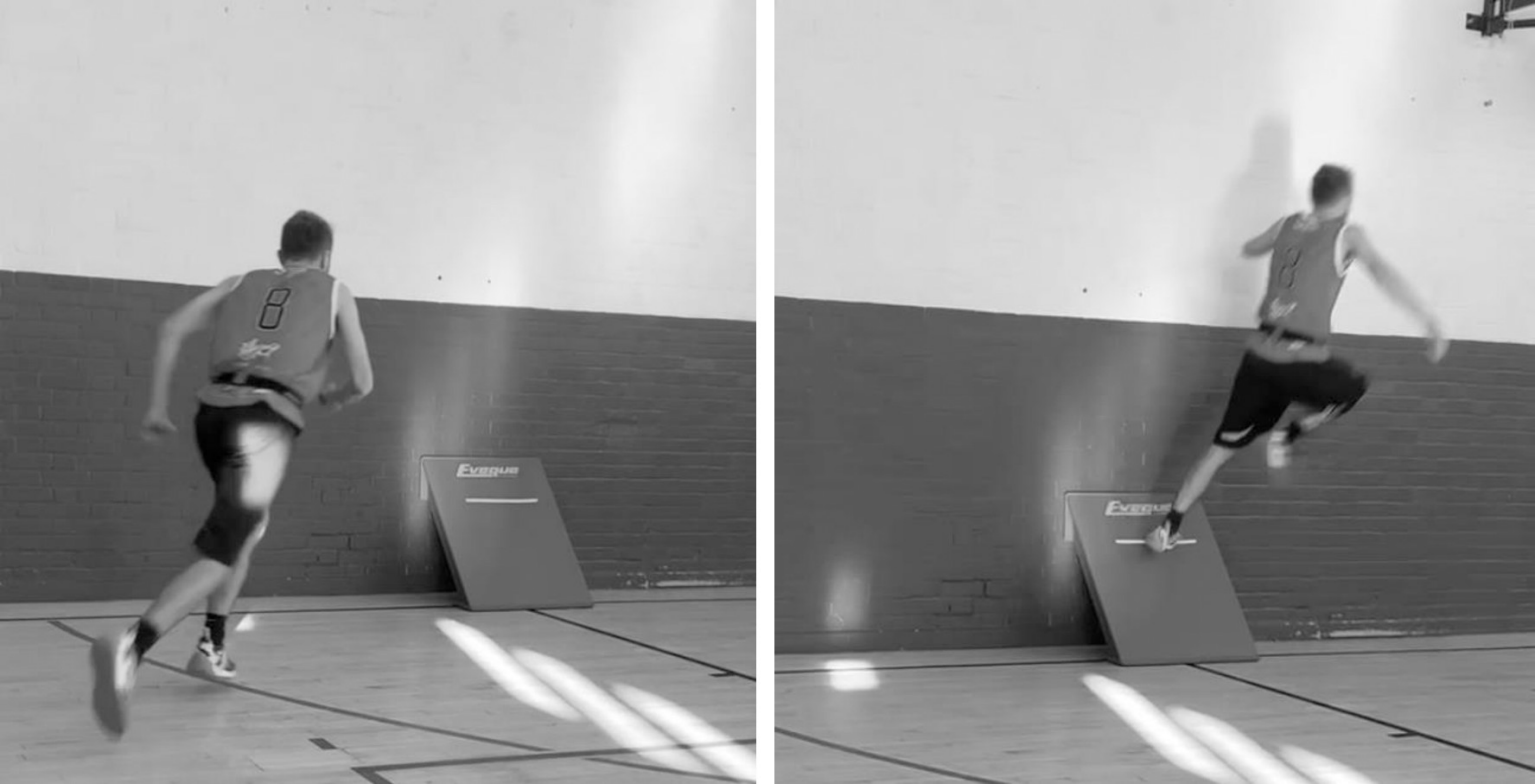
The tic tac action showing both the approach (left) and push off (right) components. After the approach run, the performer leaps towards the angled wall board, pushing off with one leg to redirect their movement, aiming to land as far as possible away from the board.

The two training interventions were embedded separately within the two weekly S&C sessions delivered as part of the players’ typical training between the months of September and November. These months constitute the first half of the competitive season. Sessions took place on Mondays and Thursdays across the 8-week period and were approximately 70-minutes in duration. Ahead of each session, participants were required to complete a warm-up comprised of whole-body mobility and activation exercises. The S&C programmes (Table 1) for both groups were matched for exercises, and prescribed sets and repetition ranges except for the exercise paired with the ‘strength exercise’ to form the ‘complex pair’. The strength exercises utilised in the complex pairs were implemented based on a progression system to ensure safe and effective execution. Specifically, where appropriate, some participants began with the goblet squat and then progressed to the front squat. This strategy ensured that participants received a strength stimulus in the squat pattern while attaining greater skill over the 8-week intervention period. However, for the second strength exercise, the hexagonal bar deadlift, no such progression was necessary. Nonetheless, each strength exercise was performed with the same coaching cue of “control down and explode up”. Immediately following the strength exercise, the TT group were required to complete a parkour-style tic-tac jumping action (Fig 1) previously described in Williams et al. [42], while the DJ group were required to execute the drop jump exercise from a box with a height of 60 cm. Following completion of the complex pair, two minutes passive rest was prescribed. The volumes for both intervention jumps were matched across the 8-week period.

**Table 1:** Training programme utilised across the 8-week intervention period. Exercise “B2” determined by the assigned intervention group. *Rear foot elevated.

**Session One**		
**Exercise Order**	**Exercise**	**Prescribed sets x repetitions**
A1	Triple hop	3 x 2 each leg
A2	Band assisted jump	3 x 5
B1	Front / goblet squat	1 x 10, 1 x 6, 1 x AMRAP, 1 x AMRAP
B2	Tic tac or depth jump	4 x 3 each leg
C1	Flat dumbbell press	3 x 8
C2	Chin up	3 x 6
D1	Isometric DB RFE* floating heel lunge	2 x 45 seconds each
D2	Nordic hamstring extension	2 x 5
D3	Dumbbell W to Y	2 x 12
**Session Two**		
**Exercise Order**	**Exercise**	**Prescribed sets x repetitions**
A1	Triple hop	3 x 2 each leg
A2	Band assisted jump	3 x 5 each
B1	Hexagon bar deadlift	1 x 10, 1 x 6, 1 x AMRAP, 1 x AMRAP
B2	Tic tac or depth jump	4 x 3 each leg
C1	Landmine ½ kneeling single arm press	3 x 8 each arm
C2	Dumbbell split squat	3 x 10 each leg
D1	Inverted row	2 x 10
D2	Dumbbell staggered stance RDL	2 x 10 each leg

To create equivalence in the loads used for the strength exercises within the complex pairs, the autoregulatory progressive resistance exercise (APRE) method was utilised. Previously described by Mann et al. [43], the APRE method provides a parameterised form of autoregulation that enables the individual to adjust training loads to account for their strength capabilities on a given training day. In the present study, the six-repetition maximum (6RM) protocol outlined by Mann et al. [43] was utilised (Table 2), which required the participant to complete four sets of the strength exercise, first performing 10 repetitions at a load approximating 50% of their anticipated 6RM, followed by a set of six repetitions at approximately 75% of 6RM. For their third set, the participant completed as many repetitions as possible (AMRP) using 100% of their anticipated 6RM. The final set of the strength exercise required the participant to complete AMRP with an adjusted load to set three using the adjustment guidelines displayed in Table 2. The same adjustment guidelines were then utilised to determine the initial loads utilised for the subsequent training session, with all volume-load for the exercise being logged by each participant on an online strength training platform. In all other exercises in the programme, loads were self-selected by the participants with guidance from the third author of the study, who was also the strength and conditioning coach supervising the programme delivery.

**Table 2:** APRE protocol for 6RM and load adjustments for set 4.

Repetitions	Intensity (% of 6RM)
APRE protocol for 6RM	
10 x	50%
6 x	75%
Maximum	6RM
Maximum	Adjusted weight
Repetitions for set 3	Set 4 adjustment (kg)
6RM routine adjustment	
0–2	-2.5 to -5
3–4	0 to -2.5
5–7	No change
8–12	+2.5 to +5
>13	+5 to +7.5

### Testing procedures

All testing was carried out by the third author and took place within the basketball academy’s usual S&C training venue. Testing was administered one week prior and one week post the 8-week intervention period and, on each occasion, across two days. In each instance, the testing took place at a similar time of day (late afternoon). On both occasions, participants completed a standardised warm-up, which consisted of dynamic stretching exercises and sprint-based running drills, and short sprints (10-20-m) that progressively increased in intensity. Following the warm-up the participants performed the test battery comprised of a 20-m linear sprint (with splits of 0-5-m, 5-10-m, and 10-20-m), the 5-10-5 *“Pro agility test”*, countermovement jump (CMJ), squat jump (SJ), the 10–5 hop test (HT), and the isometric midthigh pull (IMTP). Anthropometric measures of stature and body mass were also recorded.

The speed tests were recorded using an electronic timing-gates (Smart Speed, Vald Performance, Brisbane, Qld, Australia). Participants began each trial in a *two-point* stance, positioned 50-cm behind the first timing gate. They were instructed not to countermove before their first step and to sprint through the end timing gate. For the 20-m linear sprint, participants performed three trials and the average of the three trials for each of the splits were used in the analysis. For the 5-10-5 test, participants were instructed to sprint 5-m to the first line as fast as possible before turning and sprinting in the opposite direction to the far line (10-m away), before turning and sprinting back through the start/finish line. For both tests, trials were separated by at least two minutes.

The CMJ, SJ, HT and IMTP trials were all recorded on dual portable force platforms (ForceDecks, Vald Performance, Brisbane, Qld, Australia). For the CMJ, participants were required to jump with their hands placed upon their hips and instructed to descend to a self-selected countermovement depth before immediately jumping as high as possible. Three trials were completed with at least 20-seconds. Means of jump height (flight time), peak concentric force, relative peak concentric force, and eccentric impulse of the three jumps were used in the analysis.

For the SJ, similar procedures previously outlined by Petronijevic et al. [44] were followed. Accordingly, each participant was required to descend to a self-selected depth where they were required to hold the position for three seconds before jumping as high as possible whilst maintaining their hands being placed on their hips. A total of three SJs were completed with at least 20-seconds between trials and the average of three jumps was analysed. For the HT, following an initial countermovement jump, participants were required complete ankle dominant 10-hops, with the aim of achieving as much height as possible in each hop whilst minimising ground contact time. A total of two trials were completed by each participant with approximately two-minutes between. The reactive strength index (RSI) (calculated by the division of jump height and respective ground contract time) of the best five jumps was used for the analysis.

For the IMTP, a *power rack* setup was utilised, with the barbell set so that it was immovable by the participant. The bar was positioned at a height approximating mid-thigh height of the participant, with a knee angle between 135–145˚, and a hip angle of between 140–150˚. Using lifting straps to reduce the influence of grip strength as a limiting factor, each participant adopted a position with their shoulders slightly in front of the bar and directly over their hands, similar to the second pull of the power clean. After a weighing period of three seconds, with limited pre-tension, each participant was instructed to *“pull as hard and as fast as possible”* against the immovable bar for five seconds. A total of two trials were completed by each participant with a rest period of at least two-minutes between trials. The average of peak force from the two trials was used for the analysis.

### Statistical analysis

Statistical analyses were performed using the statistical analysis software, RStudio for Windows, version 2024.04.02. Volume loads between the two groups were analysed using an independent t-test. In relation to the pre-post physical performance measures, all data were initially tested for normality using the Shapiro-Wilk test and an intraclass correlation coefficient (ICC) was used to assess the reliability of the measurements within-subjects. To evaluate the effects of group and time on the performance measures, a generalised linear mixed model (GLMM) with a Gamma family and a log link function was fitted to the data. The model included random effects for subject and measurement to account for repeated measures and variability across different tests. Following this, R-squared values were computed to evaluate the proportion of variance explained by the fixed and random effects in the model.

In addition, effect size (ES) using pooled standard deviations were calculated to compare both within-group pre- to post-intervention measures and between-group post-intervention measures. In both cases, the ES values were interpreted as ‘small’, ‘medium’, and ‘large’ in accordance with Cohen’s guidelines [45].

## Results

The means of the load-volumes from the 8-week training intervention for the two strength exercises used in the complex training were 24371.50 ± 10426.37 kg for the DJ group, and 30458.88kg ± 7802.64 kg for the TT. The independent t-test did not reveal differences between the two load-volumes to be significant (*p* > .05).

Pre- and post-intervention descriptive data are shown in Table 3. The fixed effects of the GLMM revealed no statistically significant differences between the DJ and TT groups (Estimate = 0.095, SE = 0.075, p > .05) or between pre- and post-intervention time points across the performance measures (Estimate = 0.013, SE = 0.028, p > .05). The random effects captured substantial variability in the intercepts across subjects (Variance = 0.019, SD = 0.138) and measurement types (Variance = 5.459, SD = 2.336). The intraclass correlation coefficient (ICC) was 0.995, indicating that 99.5% of the variance in measurements was attributable to differences between subjects. The conditional R-squared value showed that the model explained 99.5% of the variance when both fixed and random effects were considered, while the marginal R-squared value indicated that only 0.1% of the variance was explained by the fixed effects alone.

**Table 3:** Pre- and post-intervention descriptive test results according to group (mean and standard deviation).

	DJ		TT	
	Pre	Post	Pre	Post
CMJ height (cm)	39.69 ± 6.82	37.91 ± 7.11	44.89 ± 9.32	42.47 ± 7.20
CMJ concentric peak force (N)	1870.86 ± 300.20	1839.86 ± 229.80	2040.43 ± 361.31	2077.71 ± 391.86
CMJ relative concentric peak force (N· kg)	24.27 ± 1.91	23.67 ± 1.81	26.17 ± 2.63	26.26 ± 2.39
CMJ eccentric impulse (N·s)	96.24 ± 22.45	95.23 ± 16.67	115.01 ± 31.92	104.89 ± 31.52
SJ height (cm)	35.13 ± 5.93	35.56 ± 7.41	39.09 ± 7.74	37.97 ± 6.60
HJ RSI (m/s)	1.22 ± 0.23	1.11 ± 0.34	1.37 ± 0.53	1.22 ± 0.41
IMTP net peak force (N)	1592.43 ± 598.88	1736.86 ± 451.22	1978.29 ± 416.23	1889.43 ± 409.72
5-10-5 change of direction speed (s)	4.98 ± 0.13	4.98 ± 0.12	5.08 ± 0.19	5.00 ± 0.16
0-10-m sprint (s)	1.80 ± 0.11	1.73 ± 0.06	1.77 ± 0.08	1.77 ± 0.08
10-20-m sprint (s)	1.30 ± 0.05	1.28 ± 0.05	1.28 ± 0.06	1.27 ± 0.06

The effect size comparisons (Table 4) revealed a small effect of group on CMJ height, with a smaller decrease in post-intervention jump height in the TT group compared to the DJ. However, the TT group were found to have a post-intervention increase in concentric peak force compared to the DJ who displayed a decrease, with a medium ES. Relative to body mass, however, the magnitude of difference in concentric peak force between the two groups was found to be small. There was a reduction in eccentric deceleration impulse between the two groups which was larger in the TT group though the ES of this difference was determined to be small. In the SJ, there was a small difference between pre-post scores, which was also reflected in the small ES between the two groups. The same outcome was also revealed for RSI comparisons for the HJ test. In the IMTP test, there was a medium ES in the pre-post peak vertical force differences between the two groups, with the DJ group displaying an increase compared to the TT group who decreased in this measure. In the sprint test, the DJ group were found to have reduced their 0-10-m time with a large ES magnitude compared to the TT group. However, this was not observed in the results for the 10-20-m split, with a small ES revealed for the post-intervention differences in performance between the two groups. Finally, for the 5-10-5 change of direction speed test, there was a decrease in time in the TT group compared to the DJ group with a small ES magnitude.

**Table 4:** Mean pre-post intervention differences and associated within group and between group Cohen’s *d* ES values according to group and physical performance test.

	DJ		TT		
Performance Measure	Mean	ES	Mean	ES	Between group ES
CMJ height (cm)	-1.77 ± 3.25	0.30	-2.41 ± 3.51	0.27	-0.19
CMJ concentric peak force (N)	-31.00 ± 117.64	0.12	37.29 ± 114	-0.24	0.59
CMJ eccentric deceleration impulse (N·s)	-1.01 ± 16.58	-0.08	-10.13 ± 23.18	0.38	-0.47
Squat jump height (cm)	0.43 ± 2.48	-0.12	-1.11 ± 5.74	0.01	-0.35
HJ RSI (m/s)	-0.10 ± 0.25	0.17	-0.15 ± 0.32	0.20	0.15
IMTP net peak force (N)	144.43 ± 393.77	-0.47	-88.86 ± 349.34	0.12	0.63
5-10-5 COD test (s)	-0.1 ± 0.18	0.08	-0.12 ± 0.29	0.45	-0.45
0–10 m sprint (s)	-0.09 ± 0.10	1.03	-0.02 ± 0.08	0.49	0.82
10–20 m sprint (s)	-0.02 ± 0.04	1.12	-0.01 ± 0.03	0.29	0.18

**Fig 2:**
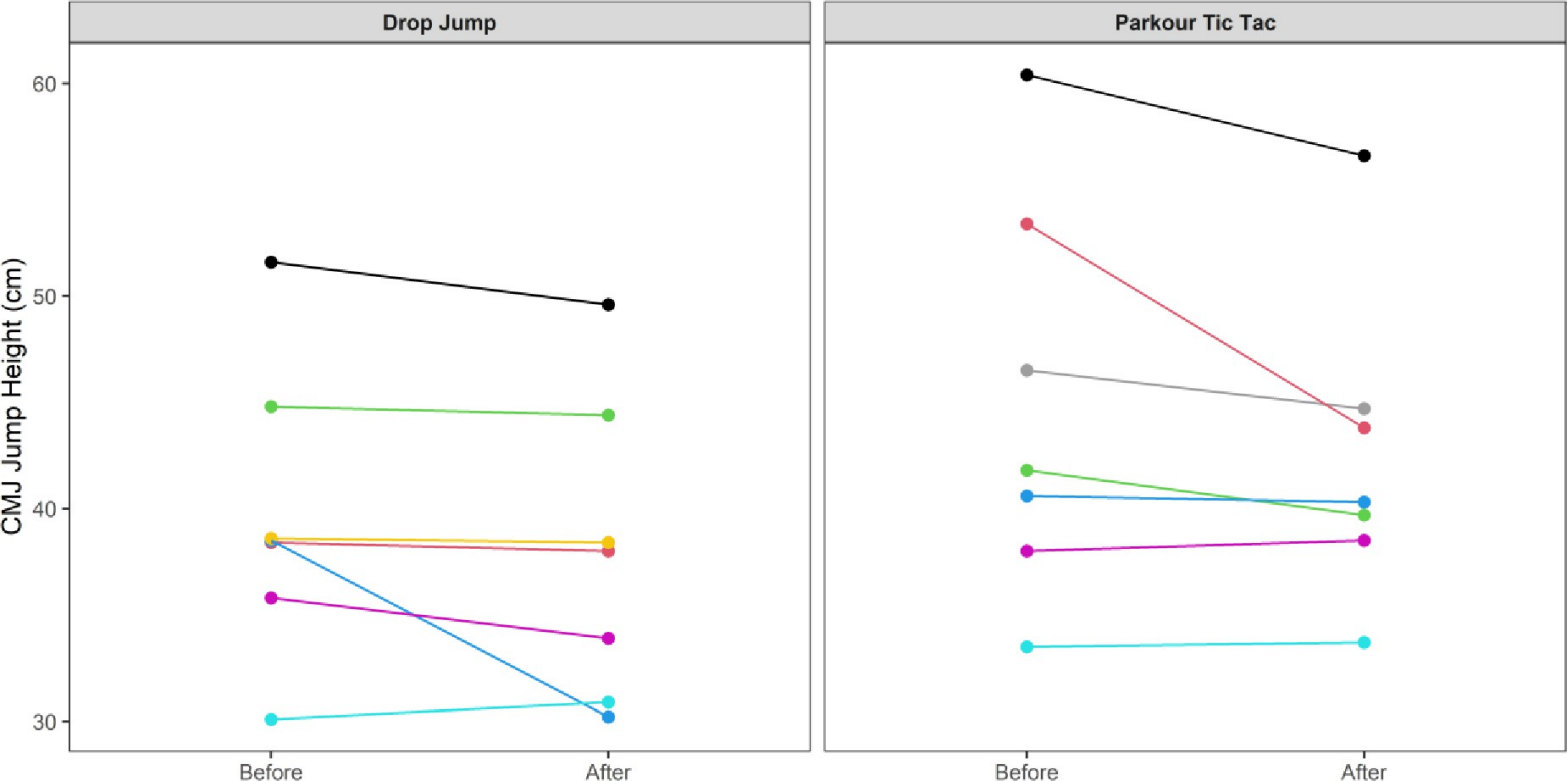
Individual mean pre-post CMJ height (cm) according to intervention group.

**Fig 3:**
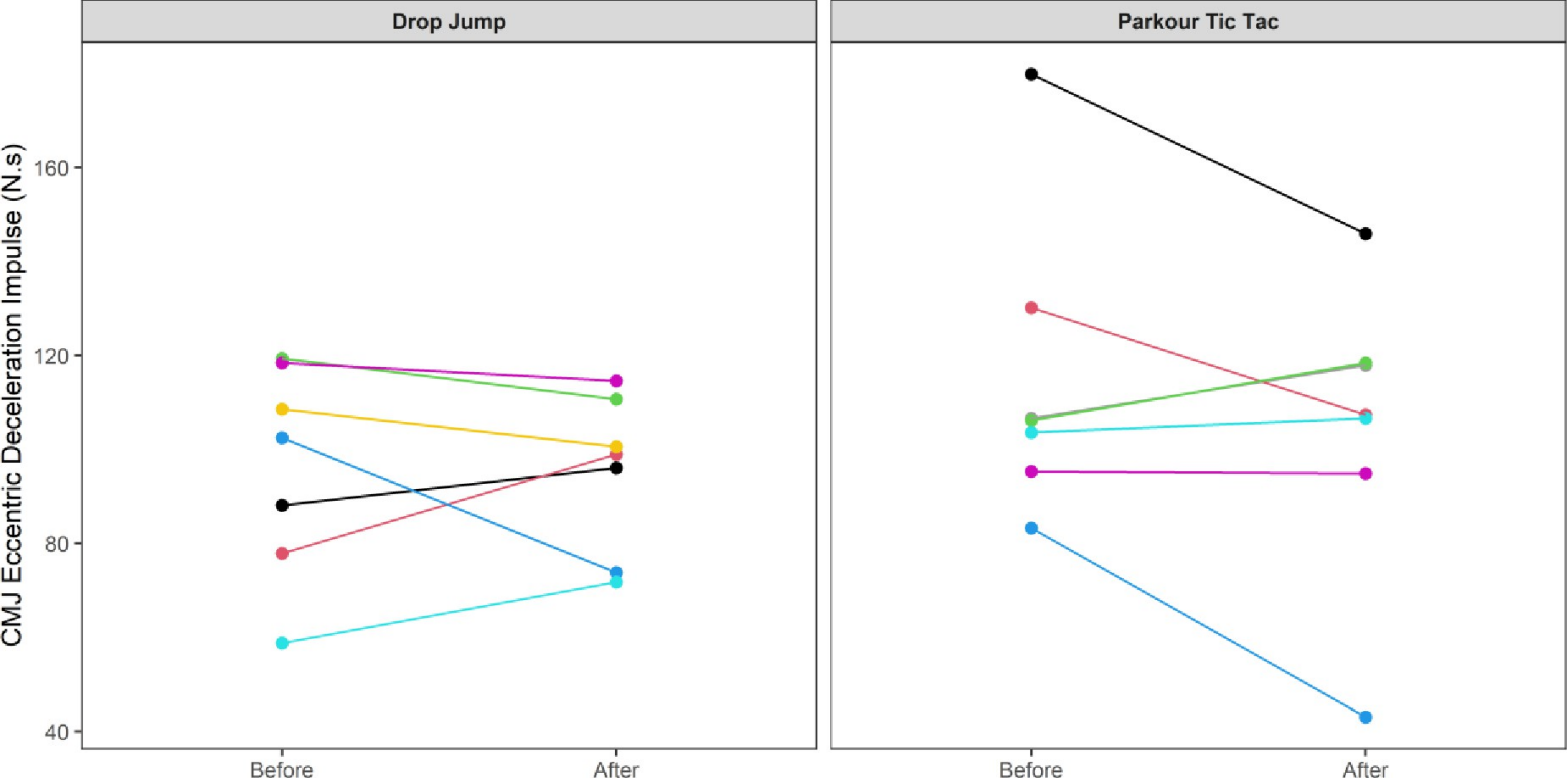
Individual mean eccentric deceleration impulse (N.s) measures from the participants’ pre-post CMJ trials according to intervention group.

**Fig 4:**
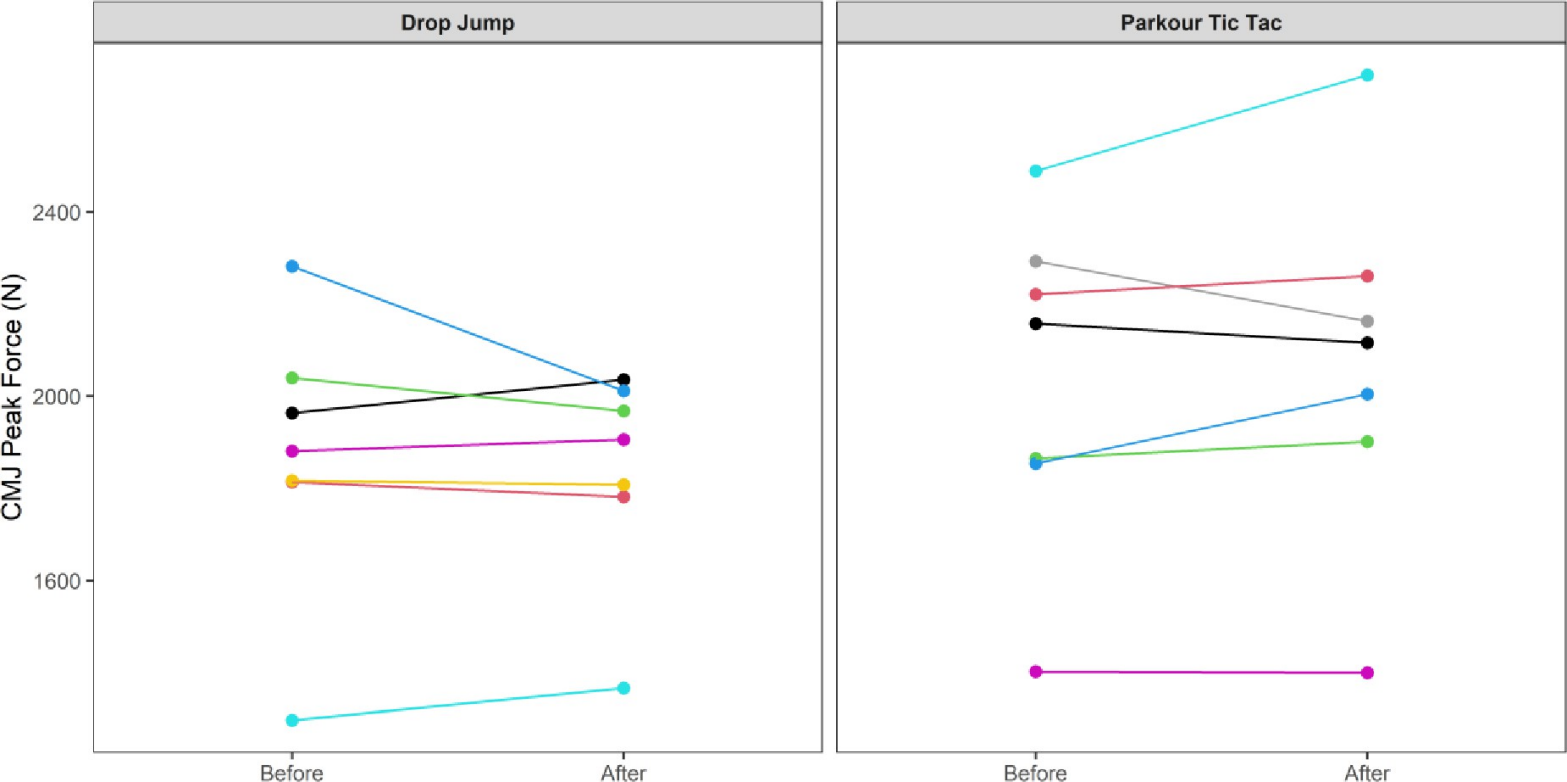
Individual mean concentric force (N) measures from participants’ pre-post CMJ trials according to intervention group.

**Fig 5:**
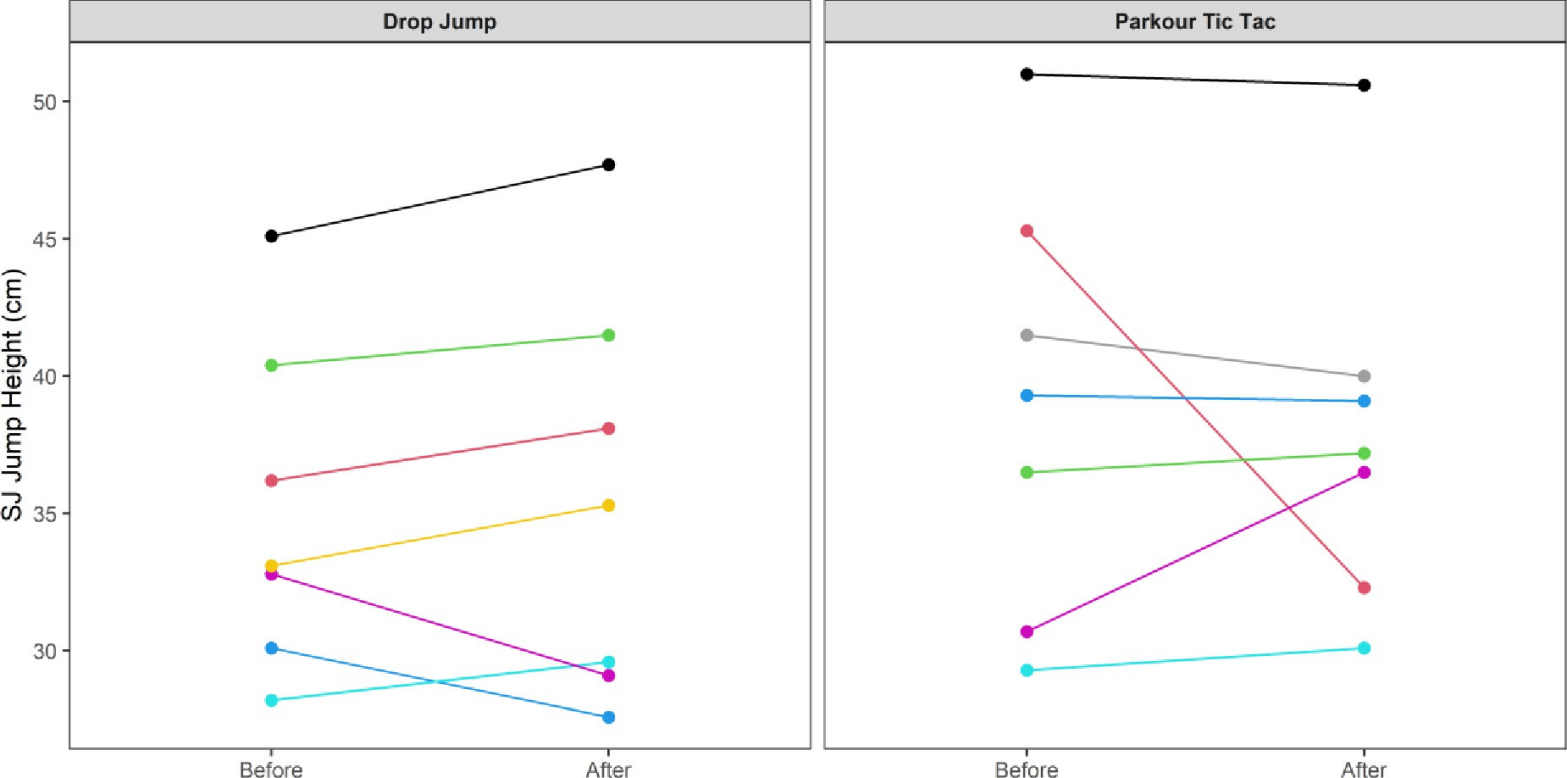
Individual mean pre-post intervention SJ height (cm) according to intervention group.

**Fig 6:**
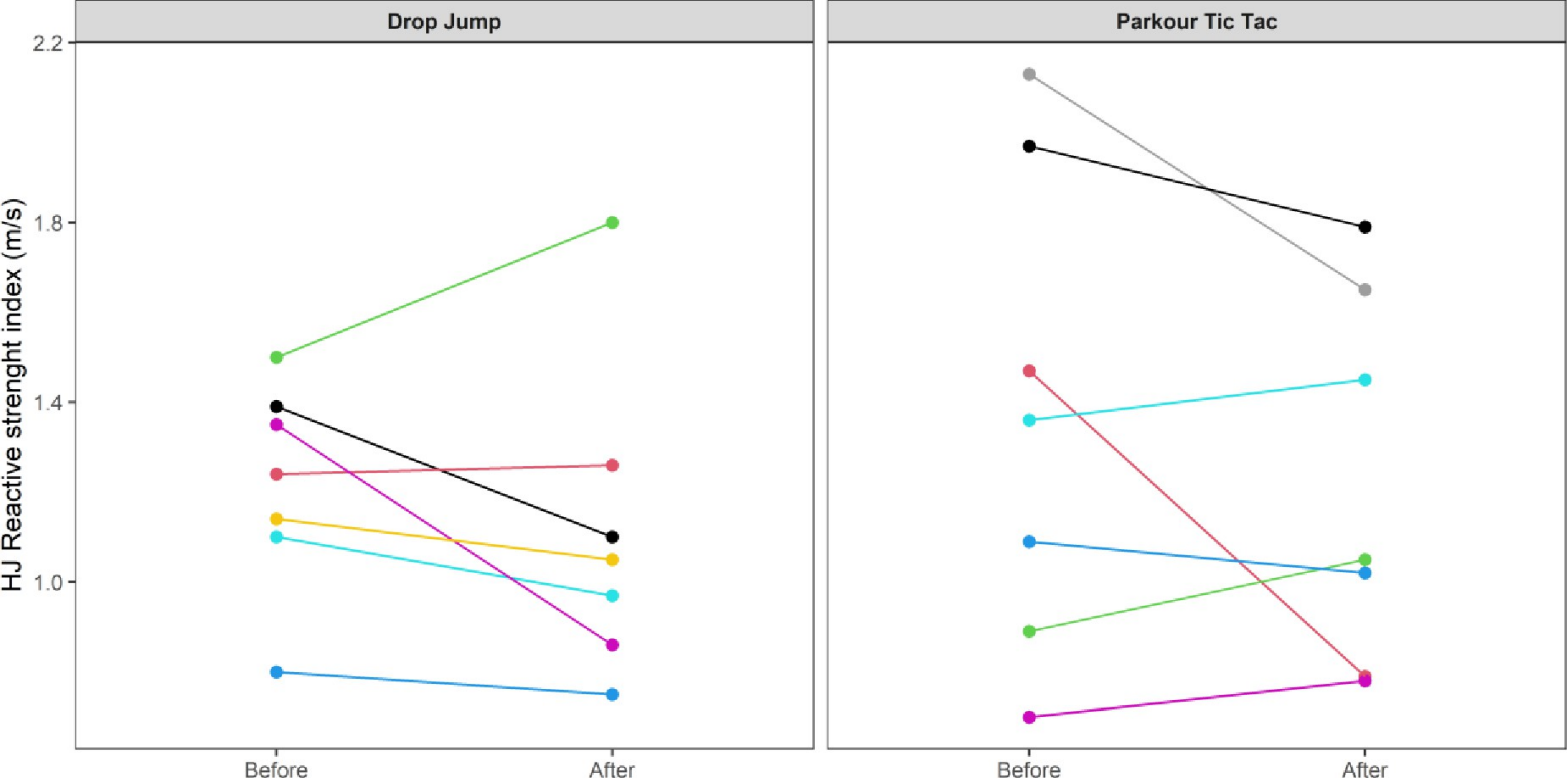
Individual mean pre-post intervention reactive strength index scores (m/s) obtained from HJ 10-to-5 test protocol.

**Fig 7:**
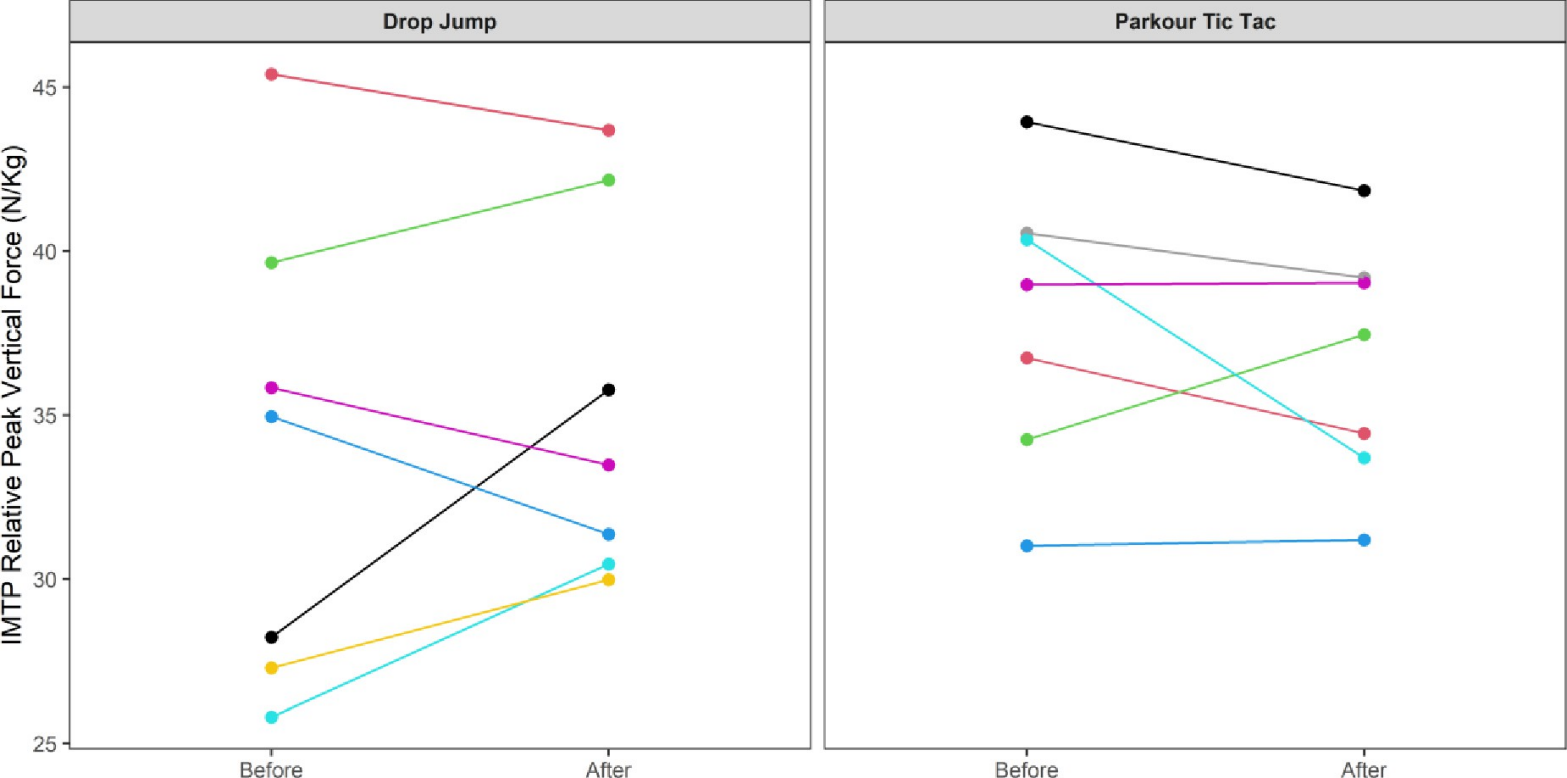
Individual mean pre-post intervention relative peak vertical force derived from the IMTP.

**Fig 8:**
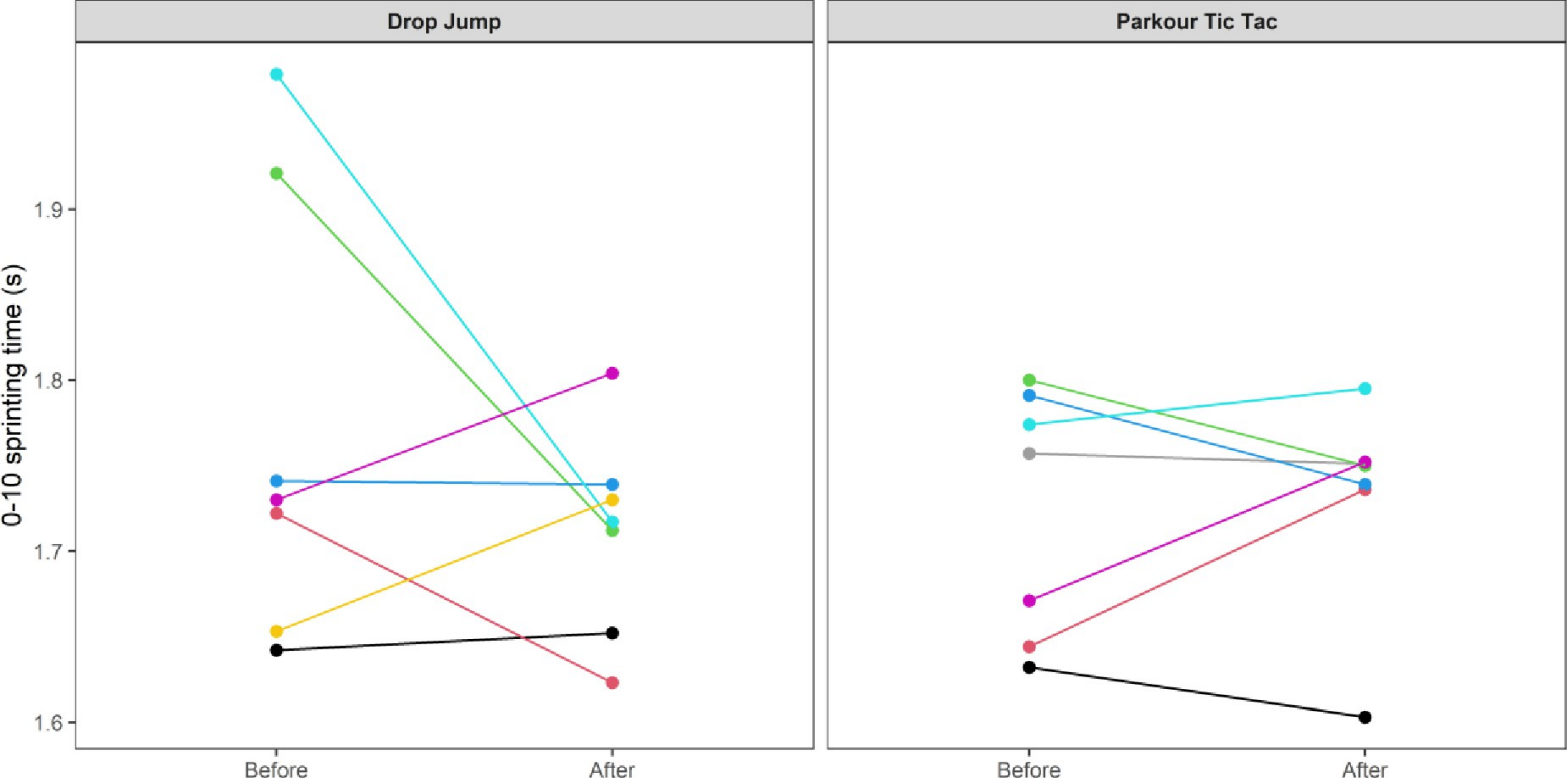
Individual mean pre-post intervention 0-10m sprint times according to intervention group.

**Fig 9:**
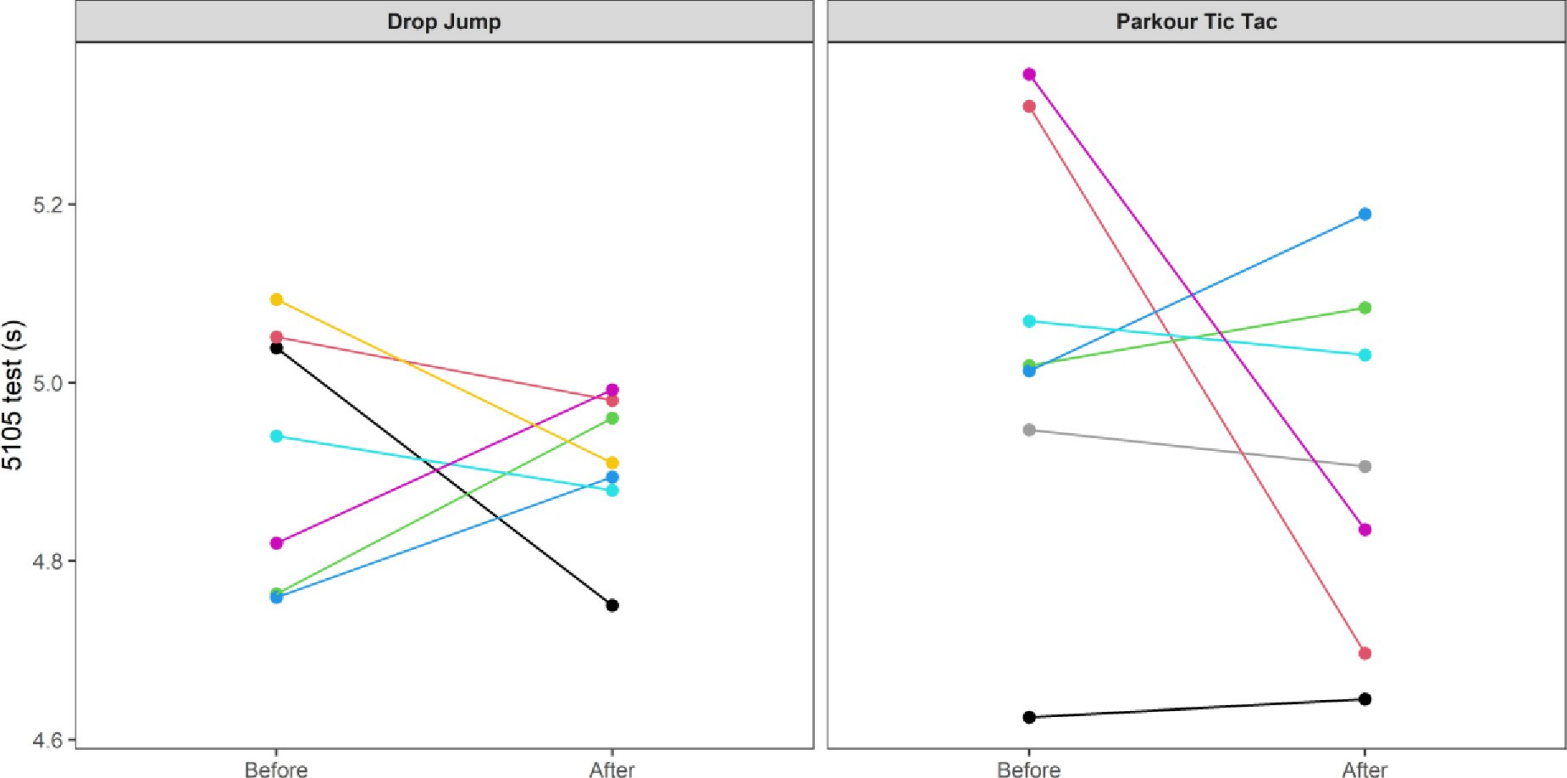
Individual mean pre-post intervention 5-10-5 change-of-direction speed times according to intervention group.

At an individual participant level, comparisons of pre-post intervention scores are displayed in Figs 2–9. Across each of the measures, there were varying levels of pre-post changes across both intervention groups, indicating an individual responsiveness to the training stimulus. Within the figures, a dashed line has been used to indicate where post-intervention scores were improved beyond an individual participant’s CV.

## Discussion

The aim of this study was to compare the effects of two different complex training interventions implemented within the normal strength and conditioning programme of talented adolescent basketball players on measures of physical performance. Although no significant differences between the two interventions were revealed, notable differences based on effect size calculations were observed. Most notably, the DJ group improved in the linear sprint measures to a larger extent than the TT group, whilst the TT group displayed greater improvement in COD speed compared to the DJ group. In jump-based measures (CMJ, SJ, and the HJ), differences appeared to be highly varied with ES that were revealed to be small. Only the IMTP test appeared to show any additional distinction between the two intervention groups, with increased force outputs observed in the DJ group only. In addition to these findings, highly individualised responses to the training interventions were observed in both groups. Therefore, while the lack of significant pre-post differences between the two interventions suggests that neither the TT nor the DJ used within complex training were effective in eliciting changes to the physical capabilities of the players, the observed nuances in the findings suggest that the training was effective, though with a high degree of variability.

The results appear to contrast with other studies [46, 47] that have investigated the effects of S&C-based training in youth basketball populations and observed significant improvements in performance-related measures. While the relatively small sample size may have accounted for the non-significant group-level results in the current study, the individualised responses to the training interventions suggest a non-linear pattern of development of physical capabilities among the youth players, despite being exposed to the same workloads. This is further supported by the high reliability of performance test measures, with most of the variance attributable to differences between subjects. In motor learning, non-linear pedagogy acknowledges the inherent complexity of motor skill development, recognising the individuality and variability in developmental patterns [48]. This approach is a central component of the ecological dynamics framework, which views the human body as a dynamic and complex system. Moreover, motor skills emerge from the interaction of individual, task, and environmental constraints, which are continuously changing [49]. Unlike traditional motor learning theories, which are often reductionist, the ecological dynamics integrates concepts from ecological psychology and dynamical systems theory to better reflect the complexity of skilled performance [50, 51]. From this perspective, the heterogenous results found in our study appear to highlight non-linear responses to the training intervention and indeed the wider S&C programme across the 8-week period. This was further highlighted by some of the participants showing improvements whilst others showed performance decrements (see the plots in Figs 2–9), indicating that responsiveness to training was non-linear and highly individualised.

The findings of the current study appear to support other research that have revealed differences between so-called *responders* and *non-responders* to training interventions [52–54]. However, from an ecological dynamics perspective, a lack of responsiveness suggests non-linear patterns of change at an individual level, rather than the categorising an individual as a non-responder. This may be especially relevant to the training exercises in this study, as the youth basketball players likely used relatively novel movement skills in which they were not yet proficient. As a result, the development of motor skill over the intervention period could have limited observable changes in post-intervention measures of physical capabilities.

From a dynamical systems perspective, Morrison and Newell [55] argue that motor learning and strength training are closely related, with the nervous system playing a primary role in early development of muscular strength before any physical changes occur. They further contend that both motor skill and strength are inextricably linked, with some tasks requiring greater contributions from one over the other. This perspective suggests that the individual responses observed in the results of this study demonstrate the human body as a complex biological system. Small changes in the performance of a motor task may lead to unpredictable system responses [56], demonstrating the non-linear, self-organising processes inherent in each individual.

For individual performers, skilled action represents the synergetic organisation of the neuromuscular system, shaped by their morphological and biomechanical constraints [57]. Accordingly, changes these constraints would have implications for motor skill performance. In relation to our findings, the individualised responses among participants likely reflect this non-linear behaviour and self-organisation, processes that are present continuously, not solely as result of the S&C intervention. For example, in response to acute levels of fatigue, the self-organising process adjust accordingly. Additionally, psycho-emotional states influence this biological system both in the short-term and across extended training periods, impacting training adaptations [58]. Our results highlight that, while the training stimuli were effective though for some participants, they appeared detrimental for others, underscoring the complex biology of the human body and the need for individualised training approaches—even in youth level athletes who are typically considered to be highly responsive to S&C-based training [59].

From the perspective of traditional S&C-based training principles, the lack of significant differences resulting from the two interventions was somewhat surprising given the relatively low training experience of the participants. Indeed, in contrast to the SAID principle, which holds that specific adaptations will occur in response to the type of training utilised [60], athletes with a low S&C training age have previously been found to improve performance despite the use of non-task specific exercises [26, 31]. For example, Latorre Román et al. [31], who also utilised complex training in youth basketball players, observed significant changes across all measures, including sprinting and change of direction speed. In contrast to the current study, however, the participants in the Latorre Román et al. [31] study were pre-PHV, which may have accounted for their responsiveness to the plyometric training. To explain this, the authors suggested that neural adaptations in the prepubertal participants resulting from the plyometric training may have increased the neuromuscular capabilities (e.g., motor unit activation, intermuscular coordination) that are transferable to sprinting and change of direction speed tasks. However, in the meta-analysis by Ramirez-Campillo et al. [61] that looked at the effects of plyometric training on physical fitness capabilities of young basketball players, significant improvements were revealed in older players (>17.15 years of age) compared young players in measures of horizontal jump distance, linear sprint and change of direction times. Concerning the SAID principle, Ramirez-Campillo et al. [61] highlighted that 27 of the 32 studies included in their meta-analysis included a combination of horizontally and vertically oriented jumps, which the authors suggested may have had relevance to the sprinting and change-of-direction speed tests, where horizontal force application is important. Indeed, Moran et al. [62] previously found that horizontally-oriented jumps are superior to vertically-oriented jumps in enhancing horizontal performance, such as short-distance sprinting. Similarly, a study by Gonzalo-Skok et al. [47] revealed specific adaptations in response to the training of specific force vectors. Therefore, it may be that the plyometric-based exercises utilised in the studies included within the meta-analysis Ramirez-Campillo et al. [61], were more specific to the performance measures than the TT and DJ in the current study.

Despite not reaching statistical significance, the within-group differences observed in the current study indicates that the two interventions may have induced their own unique adaptations. For example, the 0–10 m and 10–20 m sprint times revealed large effects from the DJ intervention compared to the TT, while the TT showed greater within-group effects on the pre- to post-times for the 5-10-5 test compared to the DJ group. In the case of the TT, it is plausible that the intervention may have contributed to improved COD movement skills that benefitted performed in the 5-10-5 test. Although it is likely that the participants’ momentum at the point of contact with the angled wall board during the TT was lower compared to their momentum during the changes of direction in the 5-10-5 test, a common strategy for developing COD skills is to practice at low intensities before progressing to high intensities [63]. On this basis, the TT may have developed COD skills, including the orientation of body segments to produce a braking impulse followed by a subsequent propulsive force in a new direction [64]. Recent research by Williams et al. [42] demonstrated that the propulsive effort in the TT action resulted in significantly greater maximal acceleration compared to the DJ in adolescent basketball players. This suggests that the TT may enhance propulsive capabilities, which potentially transferred to the 5-10-5 test. However, the Williams et al. [42]study solely focused on maximal resultant acceleration and did not include kinetic variables, leaving the specific mechanisms underlying the observed improvements in COD performance unclear.

Another consideration in relation to the lack of observed pre-post changes in the performance measures in our study relates to the complex training method utilised. Typically, complex training combines maximal or near maximal muscle actions to recruit higher threshold motor units ahead of a subsequent plyometric or ballistic exercise [30]. For example, in the previously mentioned study by Latorre Román et al. [31], an isometric half squat was combined with a subsequent plyometric activity, which may have yielded a greater PAP effect than was attained through an isotonic hexagon bar deadlift and goblet / front squat exercises utilised in our study. Although the APRE method was deemed to be appropriate to create equivalence in the training loads of the participants in the current study, who have varying levels of S&C competency and training ages, it may not have been sufficient to ensure sustained recruitment of higher threshold motor units until the penultimate and final sets of the prescribed exercises. Further, with both sets being completed to volitional failure, it is possible that rather than stimulating a PAP effect on the subsequent jumping actions, the two sets of the strength exercises induced levels of fatigue that attenuated the participants’ performances in the paired jumps. Recently, reductions in jump height and RSI in the unilateral single leg jump were found to an acute response to fatigue induced by the 30–15 intermittent fitness test in elite female basketball and handball players, aged 14–18 years [65]. Despite the utilisation of the running-based test to induce fatigue, due to repeated acceleration and deceleration phases, coupled with increasing levels of speed at each stage, the 30–15 intermittent running test is understood to place considerable demand on the neuromuscular system [66]. Accordingly, similarities in the neuromuscular demand induced by the APRE protocol and the final stages of the running test are plausible. However, prior to the two sets of the strength exercises completed to failure, irrespective of any PAP effect, two sets of the jumping actions were performed in the absence of such levels of fatigue which, alone, do not appear to have elicited any changes in physical performance. It may be, therefore, that the volume of jumps performed in each session across the 8-week training period was not sufficient to elicit changes to force characteristics of the lower limb that might have led to improved performance in the post-intervention measures. Indeed, in post-PHV males, higher jump volumes were superior to moderate jump volumes (240 jumps per week vs. 120 jumps per week) at eliciting across changes to drop jump and linear sprint performance following a seven week intervention period [67]. In contrast, the current study used substantially lower jump volumes which may have accounted for the non-significant findings. Moreover, it is possible that the magnitude of the training stimulus of the S&C programme was not greater than that experienced by the basketball-specific workloads. Given the players were part of a full-time basketball programme that required them to practice four to six times per week, undertaking one to two competitive games, their associated levels of conditioning, through exposure to high-intensity efforts, including jumping, repeated sprinting, and high-frequency changes of direction, may have warranted a greater training stimulus from the S&C programme. Indeed, the total number of plyometric sessions in a programme has previously been found to have a significant positive effect on jump height in basketball players [61], indicating that the total number of training sessions within our intervention may not have been sufficient to induce such an effect.

In addition to above, a study by Arede et al. [46], which revealed significant improvements in physical performance measures in youth basketball players in response to a 10-week strength training programme, utilised only two exercises (bench press and parallel squats) with training volumes of five sets of five repetitions and loads that optimised power output for each repetition. Accordingly, it is indeed possible that the programme used within the current study included more exercises than was optimal for positive adaptations to occur, meaning that basketball S&C coaches might need to explore a variety of different ways to successfully incorporate elements of parkour into their programming repertoires. Indeed, the supposition here appears to be further vindicated by the lack of significant change across both groups in response to other components of the training programme, particularly in relation to exercises that targeted the lower limb (e.g., the isometric DB RFE floating heel lunge).

To some extent, our results raise questions about the intended purpose of the S&C program in the development of youth athletes. Despite the benefits of S&C-based training and the scientific literature emphasizing its importance within the long-term strategy for young athletes [3, 22, 68], the effectiveness of such training may be hindered when considered against other demands, such as sport-specific training and competition. Moreover, it may simply constitute the addition of extra work for the young athlete without necessarily providing the intended value. Accordingly, it is a necessary analysis of the purpose of S&C within youth athletic development to better inform talent development processes. Where typically, the purpose of such training is to ensure the appropriate development of the physical capabilities necessary for sport, this must be balanced against the total workloads applied to the athlete and the methods of recovery to optimise adaptations [69].

The ASM, which is a model of athletic development that is based upon concepts from the ecological dynamics framework, aims to develop diverse movement capabilities that are adaptable (as opposed to fixed) and, in accordance with the concept of interacting constraints (individual, task, environment), enable the performer to develop more effectively for the complex demands of sport [39, 40]. Based upon ideas these ideas, parkour has been proposed as a method of alternative activity for the athletic development of team sport athletes [36]. The diverse movements that characterise parkour, coupled with the encouragement to explore action capabilities, have contributed to the proposed use of the activity in the athletic development of youth basketball players [41]. However, it is important to also indicate that parkour is a complementary activity to traditional S&C training and the results of this study do not support a reduction in the latter from the regular regimes of youth players. Previously, a study by Williams et al. [41], which compared a parkour-based neuromuscular warm-up to a conventional neuromuscular warm-up in pre-adolescent basketball players, found no significant differences between the groups following the 8-week intervention, although it is possible that a longer study duration may have yielded different results. Nonetheless, despite no significant differences, in a similar fashion to the results of our study, there were clearly positive individual responses to the training interventions, with little ultimate difference in their relative effects. This appears to support the non-linear and complex nature of athletic development in youth populations and the need for S&C practitioners to acknowledge the individuality of each young performer in their programming and long-term preparation strategy.

## Conclusions

The preparation of athletes in the context of team sports involves numerous challenges, particularly the need to develop many aspects simultaneously despite time constraints that can interfere with programming efficiency. Importantly, athletes operate in sometimes unpredictable and chaotic scenarios that place demands on physical movement, spatial awareness and cognitive evaluation of a given scenario. Accordingly, S&C programmes must promote all the capabilities that are required for competitive success in such scenarios. In this way, including strategies that stimulate movement variability, such as parkour, can facilitate the development of adaptable movement capabilities. In addition to other studies that may be carried out in the future to better understand the real effectiveness of this method, with the present results, practitioners are encouraged to adopt alternative strategies, such as the TT movement, that can concurrently target different aspects of physical preparation relevant to the demands of the sport of basketball.
